# A Discrete Time Model for the Analysis of Medium-Throughput *C. elegans* Growth Data

**DOI:** 10.1371/journal.pone.0007018

**Published:** 2009-09-15

**Authors:** Marjolein V. Smith, Windy A. Boyd, Grace E. Kissling, Julie R. Rice, Daniel W. Snyder, Christopher J. Portier, Jonathan H. Freedman

**Affiliations:** 1 SRA International, Durham, North Carolina, United States of America; 2 Biomoleclular Screening Branch, National Toxicology Program, Research Triangle Park, North Carolina, United States of America; 3 Biostatistics Branch, National Institute of Environmental Health Sciences, National Institutes of Health (NIH), Research Triangle Park, North Carolina, United States of America; 4 Laboratory of Molecular Toxicology, National Institute of Environmental Health Sciences, National Institutes of Health (NIH), Research Triangle Park, North Carolina, United States of America; Massachusetts General Hospital/Harvard Medical School, United States of America

## Abstract

**Background:**

As part of a program to predict the toxicity of environmental agents on human health using alternative methods, several *in vivo* high- and medium-throughput assays are being developed that use *C. elegans* as a model organism. *C. elegans*-based toxicological assays utilize the COPAS Biosort flow sorting system that can rapidly measure size, extinction (EXT) and time-of-flight (TOF), of individual nematodes. The use of this technology requires the development of mathematical and statistical tools to properly analyze the large volumes of biological data.

**Methodology/Principal Findings:**

Findings A Markov model was developed that predicts the growth of populations of *C. elegans*. The model was developed using observations from a 60 h growth study in which five cohorts of 300 nematodes each were aspirated and measured every 12 h. Frequency distributions of log(EXT) measurements that were made when loading *C. elegans* L1 larvae into 96 well plates (t = 0 h) were used by the model to predict the frequency distributions of the same set of nematodes when measured at 12 h intervals. The model prediction coincided well with the biological observations confirming the validity of the model. The model was also applied to log(TOF) measurements following an adaptation. The adaptation accounted for variability in TOF measurements associated with potential curling or shortening of the nematodes as they passed through the flow cell of the Biosort. By providing accurate estimates of frequencies of EXT or TOF measurements following varying growth periods, the model was able to estimate growth rates. Best model fits showed that *C. elegans* did not grow at a constant exponential rate. Growth was best described with three different rates. Microscopic observations indicated that the points where the growth rates changed corresponded to specific developmental events: the L1/L2 molt and the start of oogenesis in young adult *C. elegans*.

**Conclusions:**

Quantitative analysis of COPAS Biosort measurements of *C. elegans* growth has been hampered by the lack of a mathematical model. In addition, extraneous matter and the inability to assign specific measurements to specific nematodes made it difficult to estimate growth rates. The present model addresses these problems through a population-based Markov model.

## Introduction

To predict the toxicity associated with human exposure to potential toxicants; the National Toxicology Program, the U.S. Environmental Protection Agency and the National Institutes of Health Chemical Genomics Center are developing methodologies for the acquisition and analysis of high- and medium-throughput screening data from *in vitro* and alternative *in vivo* systems [1]. As part of this program, several *in vivo* toxicity assays have been developed which use *Caenorhabditis elegans* (*C. elegans*) as a model organism [2].

*C. elegans* is a small (∼1 mm) free-living nematode, which may be one of the most thoroughly studied metazoans in terms of its cell biology, genetics, development, and behavior. One of the strengths of *C. elegans* as a model organism is the high degree of evolutionary conservation in its biological processes [3]. *C. elegans* develop from fertilized egg to gravid adult in about 3 days at 20°C [4], [5]. A single adult hermaphrodite has the ability to produce approximately 300 offspring. Offspring mature through four distinct larval stages, L1–L4, growing in spurts between stages after molting old cuticles. Growth of *C. elegans* is approximately exponential through the larval stages [6], [7].

To study the effects of chemical toxicants on *C. elegans'* biological processes (e.g., growth, feeding, fecundity) in medium- and high-throughput fashion, a collection of tools in robotics, and image acquisition and analysis have been developed [8]. A key tool for *C. elegans*-based toxicological assays is the COPAS Biosort flow sorting system [9]. The Biosort is capable of rapidly measuring size and fluorescence parameters of individual nematodes. It can then sort and dispense nematodes based on those parameters. The Biosort has the ability to acquire optical measurements of hundreds of nematodes per minute as they flow past laser beams in the flow cell. As nematodes pass through the flow cell axial length, extinction, and fluorescence are simultaneously measured using a pair of lasers. The interruption of the laser is recorded as a pulse. The length of the pulse is defined as time-of-flight (TOF), while the total area under the pulse is recorded as optical extinction (EXT) [9]. Both TOF and EXT are indicators of the size and age of an individual nematode; however, at this time there is no direct conversion of EXT or TOF measurements to physical units of length or mass. TOF and the EXT increase as *C. elegans* develop from larvae to adults.

Some of the fundamental biological processes affected by toxicant exposures are growth and development. The use of high- and medium-throughput technologies requires the development of mathematical and statistical tools to properly analyze the large volumes of biological data acquired during a *C. elegans* growth study. To assess the effects of potential toxicants on *C. elegans* growth, a quantitative model has been developed to estimate frequencies of log(EXT) and log(TOF) measurements. Optimized model parameters are then used to compute estimated growth rates, with respect to both log(EXT) and log(TOF). In addition, this model is useful in estimating the numbers of measurements on detritus versus nematodes.

## Methods

### Data and Experiment Description

A 60 h growth assay was performed to develop a mathematical model using the Bristol N2 strain of *C. elegans*. To prepare assay samples, nematodes were maintained at 20°C on K-agar plates seeded with *E. coli* OP50 [10], [11]. Embryos were then prepared by bleaching and larvae were allowed to hatch in the absence of food to generate age-synchronized populations of nematodes, as previously described [12]. When L1 nematodes are starved their development arrests, then once food becomes available the nematodes grow as a synchronized population [13]. After hatching, starved L1s were transferred to the sample cup of a COPAS Biosort (Union Biometrica Inc., Somerville, MA, USA) and diluted to approximately one nematode/µL in complete K-medium (51 mM sodium chloride, 32 mM potassium chloride, 3 mM calcium chloride, 3 mM magnesium sulfate, 13 µM cholesterol) [8], [10]. Twenty-five L1s were loaded into each well of a 96-well plate containing streptomycin-killed (1 mg/kg) *E. coli* OP50 diluted to A_550_ = 0.5–0.55 in complete K-medium. *C. elegans* were incubated without shaking at 20°C for the duration of the experiment. All COPAS Biosort measurements were performed with EXT signal gain = 80, EXT integral gain = 100 and EXT signal threshold = 80, and a TOF minimum of 10.

Five cohorts consisting of 12 wells or 300 nematodes (12 wells x 25 nematodes/well) were loaded at t = 0 h. Every 12 h, one cohort was aspirated using the Biosort and the last cohort at 60 h after loading. Both TOF and EXT measurements were acquired at loading and aspiration.

To determine the effects of *C. elegans* mobility and shape on measurement parameters, a total of 3,600 *C. elegans* were loaded into a 96-well plate and incubated at 20°C for 48 h. Fifteen minutes before aspiration, nematodes in 48 of the wells were anesthetized by adding 0.21 mM sodium azide (final concentration). The remaining nematodes were aspirated without anesthetization.

To associate log(EXT) and log(TOF) values with a specific stage of *C. elegans* development, nematodes at 25 individuals/well were aspirated after 8, 10, 26, 30, 32, 36, 50 and 52 h incubations at 20°C. An aliquot of each aspiration was also examined and the stage of *C. elegans* development determined by direct microscope examination [5].

### Model Description

The model is explicated for log(EXT) measurements. Adaptations for log(TOF) measurements are described in the Results section. Mathematical details of the development of the model and optimization are presented in the Supporting Information S1.

#### Log(EXT) Measurements

Analysis of *C. elegans* data is performed using a Markov model [14] for the growth of populations or cohorts of nematodes over time, rather than for individuals. The range of log(EXT) values is discretized into bins to define the frequency distributions of the measurements. Frequency distributions are visualized by plotting the numbers of nematodes with measurements falling in any particular bin at the right edge point of that bin and then connecting the points.

**Figure 1:**
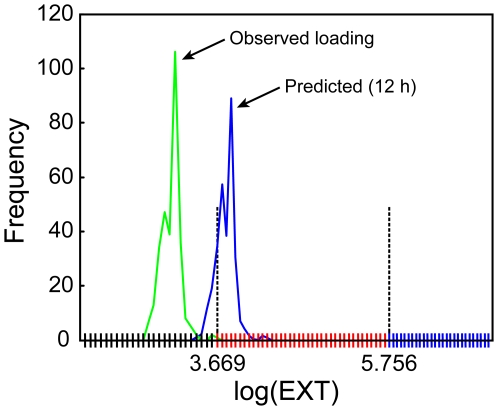
Observed loaded and predicted frequency distribution of log(EXT) measurements of *C. elegans*. The observed loading distribution for the L1 cohort (*green line*) and the corresponding prediction for the sample aspirated after a 12 h prediction (*blue line*). The horizontal axis is divided into three sections with smaller bin sizes for each section: 2.000–3.669; *black*; 3.669–5.756, *red*; 5.756–7.000, *blue*. The estimated three-valued growth rate is constant over each section.

A nematode from a particular bin at loading is assumed to grow by advancing 10, 11 or 12 bins over 12 h with respective ‘transfer’ probabilities p_1_, p_2_, or p_3_, which add to 1. This range of values and bin structure accurately describes the biological data. Changing the degree a nematode grows (e.g., 8, 9, or 10 bins versus 10, 11, or 12) or possible bin numbers to advance (i.e., 4 or 5 versus 3) does not affect the growth rate estimates. The observed frequency distribution of log(EXT) measurements made while loading a cohort of nematodes and the model output resulting from advancing each nematode in the cohort 10–12 bins according to the estimated transfer probabilities are shown in Figure 1. These predicted frequencies combined with the lognormal distribution for extraneous measurements (see below) form the estimated frequencies. The word prediction is used in this paper to refer to estimates made from the model after optimization, much as any calculated output from any model may be referred as a prediction. It does not refer to predictions made for use with other data or in different circumstances. The value of the model for the analysis in this paper lies in the estimation of growth rates of nematodes over time under specific conditions, including exposures to possible toxicants. As with any other statistical model, application to a different data set would require re-estimation.

Because *C. elegans* have been reported to develop nearly exponentially [6], the growth rate of log(EXT) was expected to be constant. However, a constant rate of growth over the duration of the experiment does not provide the best fit to the data. Even in the log scale, younger nematodes grow at a faster rate. As *C. elegans* develop the growth rate slows. Since a nematode always advances 10–12 bins with the same transfer probabilities, allowing the growth rate to change requires allowing the size or width of the bins to change: larger bins correspond to faster growth rates.

Fitting the data to the model using a single-valued growth rate (i.e., all bins have equal size) underestimates the growth at 12 h, while overestimating the growth at 60 h, which shows that a single growth rate is not sufficiently flexible. A three-valued growth rate, corresponding to three different bin sizes, allows an accurate fit at all time points and is the model used for this analysis. In Figure 1, bins marked along the horizontal axis have three different sizes separated at log(EXT) values 3.669 and 5.756, which mark changes in growth rates. The points where the bin sizes change will be referred to as change points. The larger bins (i.e., faster growth rate) have lower log(EXT) values and correspond to younger nematodes.

The estimated frequency distributions at aspiration are computed as the product of a ‘growth’ matrix, *G_EXT_*, and the empirical loading frequency distribution stacked as a vector. Such a growth matrix is defined as being a square zero matrix with three non-zero subdiagonals, starting at 10, 11, and 12 rows below the diagonal. The top subdiagonal consists entirely of the probability, p_1_, the next subdiagonal consists of the probability, p_2_, and the last subdiagonal consists of the probability, p_3_. The number of rows in *G_EXT_* is the total number of bins used to span the log(EXT) range. An example growth matrix is presented in the Supporting Information S1. In the context of Markov models, such a matrix is referred to as a probability transfer matrix.

To estimate growth for cohorts incubated over k observation intervals before aspiration, the matrix *G_EXT_* is raised to the k^th^ power. Thus, the predicted frequency distribution for data collected by aspiration after 12 k h for k = 1, …, 5, will be given by v = 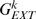
v_0_, where v_0_ is the loading vector at t = 0 and v is the vector of predicted frequencies at 12 k h. Since the range of log(EXT) values is fixed, allowing both change points and numbers of bins to either side of the change points to vary will affect bin sizes and also growth rate estimates.

The expected number of hours required for a nematode to reach a change point is computed as follows. Assume there are n_1_ bins to the left of the change point: Using the estimated transfer probabilities p_1_, p_2_ and p_3_, the time to cross any single bin in hours is 12 h/10 bins with probability p_1_, 12 h/11 bins with probability p_2_, and 12 h/12 bins with probability p_3_. The expected time to cross any single bin is then given by the following expression.
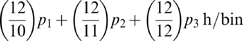
(1)


If a nematode belongs to the i^th^ bin at t = 0, then the nematode will have to cross n_1_-i bins to reach the change point. Using the loading vector and L_0_ for the total number of nematodes in the loading vector, the proportion of nematodes loaded into the i^th^ bin can be written as v_0_(i)/L_0_, so that the expected time for the cohort to reach the change point is computed as
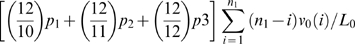
(2)


To find the estimated growth rate in log(EXT) units/h for a given section, the bin size or width in log(EXT) units for that section is computed by dividing its length (in log(EXT) units) by the estimated number of bins in the section. The growth rate estimate is the bin width in log(EXT) units divided by the average number of hours needed to cross a bin, as given in Equation {1}.

Using different bin sizes to model different growth rates forces a concomitant change in the predicted spread or range of measured outcomes. The number of bins occupied at a particular aspiration point is set by the structure of the model (three transfer probabilities) with the size of the bins determined by the estimated growth rates. If the growth rate is slow, then the bin sizes are small and the predicted spread of measured outcomes will also be small.

#### Incorporating Extraneous Measurements

**Figure 2:**
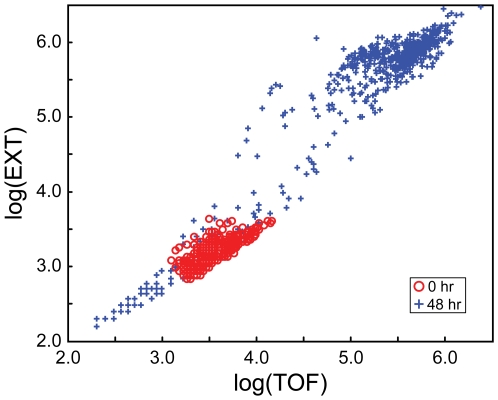
Log(EXT) and log(TOF) values of individual nematodes in the 48 h cohort. Measurements were made while loading L1 nematodes (*red circles*) and while aspirating the same nematodes following 48 h incubation (*blue crosses*). Measurements with log(EXT) and log(TOF) values higher than the loaded L1s (i.e., upper right) correspond to young adult nematodes while those with values lower than the loaded L1s (i.e., lower left) correspond to extraneous material (e.g., detritus, clumped bacteria, etc.).

Since neither birth nor death are included in the growth matrix, the predicted distribution includes the same number of nematodes as were loaded, but re-distributed over different bins. It does not predict the numbers of low-valued log(EXT) measurements seen to the left of the loaded L1s (e.g., Figs. 2–4). Microscopic examination of the low-valued log(EXT) measurements shows that they contain aspirated detritus and unhatched embryos. In the studies described in this paper focus only on the development of the initially loaded nematodes, therefore unhatched embryos and detritus are treated as extraneous measurements. Individual measurements from the Biosort cannot be associated with detritus, embryos, or nematodes. Therefore, the model includes all of the measurements. Using a skewed distribution, such as the lognormal distribution for extraneous frequencies, the following mixture is fit to all observations aspirated at time t = 12 k.
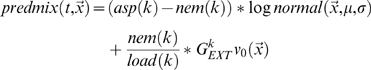
(3)


**Figure 3 pone-0007018-g003:**
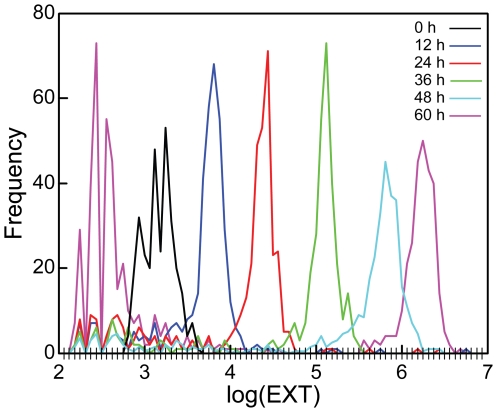
Histograms of *C. elegans* measurements at five observation times. The loading distribution of L1s (t = 0 h) from the 48 h cohort is presented to exemplify a typical loading histogram (*black line*). The total number of nematodes with log(EXT) measurements falling in any particular bin is plotted at the right edge point of that bin and then these points are connected. Extraneous observations are seen between log(EXT) 2–4 and frequency values less than 10. The large number of log(EXT) measurements between 2 and 3 for the 60 h sample include embryos.

**Figure 4:**
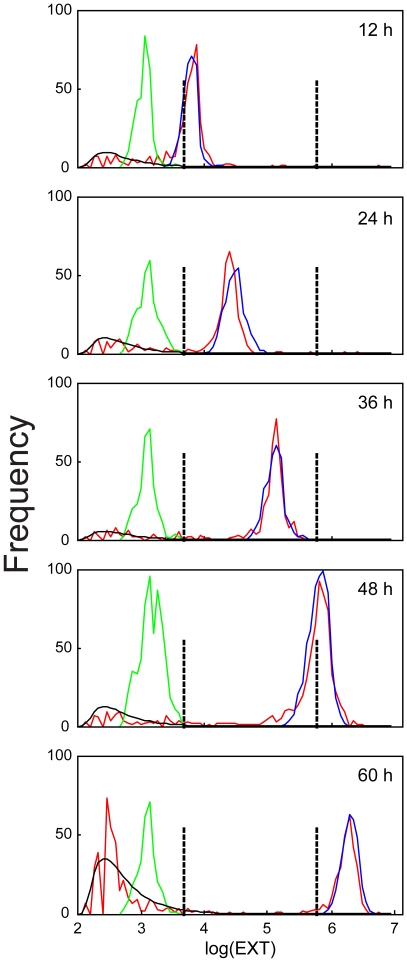
Observed and predicted frequency distributions of log(EXT) measurements at five observations times. The loading distribution for the cohort aspirated at the indicated time (*green line*) and the observed measurements (*red line*) are presented. The estimated lognormal distribution describing the extraneous measurements for each cohort (*black line*) and the predicted frequency distribution for the aspirated measurements for nematodes based on the growth model (*blue line*) are presented. The vertical dashed lines indicate the change points at log(EXT) values of 3.669 and 5.756.

Here asp(k) denotes the total number of aspirated observations (both nematodes and extraneous material) made at t = 12 k, and nem(k) denotes the predicted number of nematodes aspirated. The quantity, load(k), gives the number of nematodes loaded for the k^th^ cohort. The loading vector, 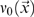, and the log normal distribution are written as functions of 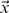, the vector of bin edges. The difference, asp(k) – nem(k), predicts the number of observations made on detritus. The matrix product in the 2^nd^ term sums to the number of loaded nematodes for that cohort, because the growth matrix does not include birth or death terms. Thus, the 2^nd^ coefficient, nem(k)/load(k), denotes the fraction of the nematodes loaded at t = 0 corresponding to the cohort of nematodes that are aspirated at t. Equation {3} describes the predicted frequencies of values observed by aspiration at time t, evaluated at the vector of bin-edges, 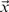. The parameters estimated by fitting the model to the observed aspirated frequencies at all five aspiration times comprise: two transfer probabilities (because the three probabilities sum to one), the numbers of bins in the three sections defined by the two change points, the change points themselves, and five estimated numbers of aspirated nematodes for the five observation points.

Although the model described above was able to adequately describe the growth dynamics of *C. elegans*, varying bin sizes created a problem when optimizing the fit of the model to observed frequencies at aspiration. The parameters of the model were estimated using least squares estimation. That is, we found the parameter values that minimize the following expression,
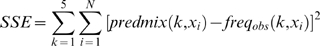
(4)where the terms were summed over the bins in the grid (i = 1,…,N) and the observation times (k = 1, …, 5). Both the mixture model and the observed frequencies in Equation {4} were evaluated at the same bin edges, x_i_. However, if the same bins that were varied to model the growth rates were used to define the observed frequencies, then the optimization problem was poorly defined. That is, as the x_i_ changes as part of the model fit, the bins change and thus the observed frequencies in Equation {4} also change. To address this problem, the curve of predicted frequencies evaluated at the variable bin edges used in the optimization were given interpolated values at the equally-sized bin edges used to define the observed observations. This solution is discussed in detail in the Supporting Information S1.

## Results

### Experimental Design and Data Acquisition

In a typical growth experiment, 25 starved L1 *C. elegans* were dispensed into each well of a 96-well microtiter plate and then incubated under the conditions defined by the experiment. At specified observation times, nematodes were aspirated from the wells and TOF and EXT measurements were acquired for each individual. Although the same nematodes were measured twice, specific measurements could not be assigned to specific individuals.

Figure 2 shows log(TOF) and log(EXT) measurements for nematode samples when L1 nematodes were loaded, and after incubating for 48 h. The 48-h measurements were concentrated in two areas: the upper right and lower left of the loading measurements. Measurements to the upper right corresponded to L4 nematodes, as indicated by larger log(TOF) and log(EXT) values. The presence of L4 *C. elegans* was also confirmed by direct microscopic examination. Measurements to the lower left corresponded to extraneous material that passed through the flow sorting system. The material in the extraneous measurements included detritus, discarded nematode cuticles, and/or clumped bacteria, based on microscopic examination. Extraneous measurements were well separated from larger nematodes (L3-adult), however, for younger animals (L1-L2) the groups may have overlapped (e.g., Fig. 3). This could become an important consideration when experimental conditions retard growth or add to the extraneous measurements (e.g., chemical precipitates).

### *C. elegans* Population Distribution

The model was applied to the 60 h growth assay in which five cohorts of 300 L1s each were loaded at t = 0 h and then one cohort was aspirated every 12 h. Figure 3 shows histograms of log(EXT) measurements at all observation times. Tick marks along the horizontal axis delineate 75 bins of equal size across the range of observed log(EXT) values. The frequency distributions moved from the 1^st^ aspiration at t = 12 h to larger log(EXT) values as nematodes grew until the final observation point at t = 60 h. One of the loading distributions of L1 nematodes (t = 0 h) is shown for reference. Microscopic observations confirmed that measurements with log(EXT) values lower than the loading distribution corresponded to extraneous measurements (detritus, discarded cuticles, etc) at the 12–48 h time points, and *C. elegans* embryos at the 60 h time point (2–3 log(EXT) units). The distinction between extraneous and nematode measurements was clear for larger nematodes. At the earliest aspiration time (t = 12 h) however, measurements were made continuously from the left of the loading distribution to the right, which made it difficult to distinguish the aspirated nematodes from the extraneous material.

### Growth Model

A Markov growth model that predicts the frequency distribution of measurements on nematodes at various aspiration times was used. The empirical frequency distribution of log(EXT) measurements of the loaded L1s and the observed and predicted counts for 12 to 60 h aspiration times are presented (Fig. 4). Extraneous measurements were modeled by a lognormal distribution that was appropriately scaled by the optimization. The empirical loading distribution was used by the model to calculate the predicted frequencies of the aspirated nematodes using the same model parameter estimates for all time points. Since individual measurements of nematodes could not be distinguished from those of extraneous matter, all aspirated measurements were fit to a weighted sum of the lognormal distribution and the predicted nematode frequencies at each time point. The area under the predicted nematode frequencies at each time point is an estimate of the number of aspirated nematodes when the model is fit via optimization (See Equation {3}). Estimated numbers of aspirated nematodes agreed well with the observed frequency and the manufacturer's estimate of 85% recovery by aspiration (Union Biometrica, personal communication).

The Markov model assumes every nematode grows the equivalent of 10, 11 or 12 bins over a 12 h period with corresponding probabilities held in common over all time points. To allow growth rates to vary, bin sizes were allowed to vary (Fig. 1). The range of log(EXT) values was divided into three sections, separated by change points at log(EXT) values of 3.669 and 5.756. The change points at 3.669 and 5.756 were shown by microscopic observations to correspond to the L1/L2 molt and onset of gravidity in young adults, respectively. Within each section the bins were of equal size; bins became smaller for sections containing larger log(EXT) values. Because the probabilities of advancing any number of bins as a nematode grew during each 12 h interval were the same, the smaller bins correspond to a slower growth rate. The number of bins within each section and the change points were found by optimization and were common to all time points in the experiment. Estimated growth rates and change points are shown in Table 1.

**Table 1:** Variation in Growth Rates during *C. elegans* Development.

Growth Rate^1^ (log(EXT) units/h)	Section^2^ (log(EXT) units)	Growth Rate (log(TOF) units/h)	Section (log(TOF) units)
0.0620	2.000–3.669	0.0759	2.000–4.035
0.0531	3.669–5.756	0.0430	4.035–5.281
0.0445	5.756–7.000	0.0281	5.281–7.000

1 Growth rates were calculated from the Markov model using log(EXT) and log(TOF) measurements.

2 “Section” indicates the regions of the population used to calculate growth rates.

The expected time taken by a nematode to reach a change point can be computed using model parameters and loading distributions. Microscopic observations can also be made at these estimated times to determine the biological events linked to growth rate changes. Growth with respect to log(EXT) units slowed down with change points at 11 and 51 h. Observations made between 8 and 12 h indicated that nematodes increased in size from loading (t = 0) to t = 8 h and continued to increase at the L2 stage. The second change point for the log(EXT) growth rate occurred around t = 51 h. Microscopic observations made at 50 and 52 h showed mostly young, non-gravid adult nematodes while observations made at 60 h indicated the presence of gravid adults and eggs.

### Log(TOF) Measurements

Directly applying the growth model to log(TOF) measurements produced poor results, compared to log(EXT) measurements. Furthermore, altering basic structure parameters of the model, such as increasing the number of transfer probabilities or allowing more change points and growth rates, did not improve the fit. When Figures 4 and 6 were compared, greater variability in the TOF measurements were observed. The poorer results were attributed to the model underestimating this greater variability. Because TOF measurements are related to nematode length, partial curling of the nematode as it passes through the flow cell could affect the value. By ascribing the increased variability in TOF measurements to curling, other parameters affecting TOF measurements (e.g., flow rate, temperature) were assumed to be constant over the duration of the experiment. The lower variability of the EXT measurement may be a consequence of the semi-transparent nature of *C. elegans* [4]. A curved nematode would block approximately the same amount of light as a straight nematode.

**Figure 5:**
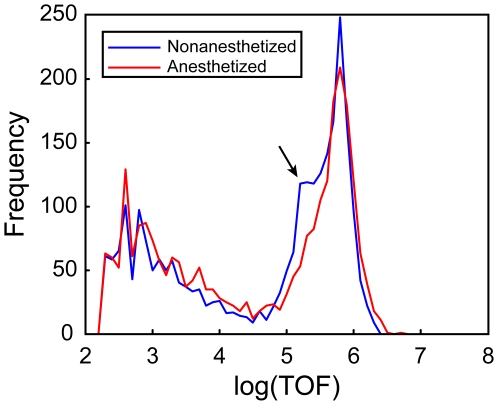
Effects of nematode shape on TOF measurements. TOF measurements were acquired for anesthetized (*red line*) and non- anesthetized (*blue line*) *C. elegans*. The ‘shoulder’ corresponding to the higher frequencies (*arrow*) near log(TOF) = 5 as well as the slight bias to the left of the non-anesthetized nematodes is consistent with some of the nematodes curling or bending while passing through the flow cell.

To determine if nematode curling was a contributing factor in TOF variability, 3,600 L1s were loaded and then aspirated following 48 h incubation. Before aspiration, half the nematodes were anesthetized. The anesthesia caused nematodes to relax allowing them to be straight as they passed through the flow cell. Results presented in Figure 5 are consistent with a portion of the non-anesthetized nematodes being curled as they flow past the lasers. The non-anesthetized *C. elegans* had a population with reduced log(TOF) values, as indicated by the small peak to the left of the majority of the measurements (maximum log(TOF) ∼5.7). This suggests that curling may be a factor in TOF variability.

The TOF measurement was defined as the length of the pulse generated by the nematode as it passed the laser. However, as indicated above, curling may lead to increased variability and could attenuate the TOF measurement without affecting the true length of the animal. Thus, growth would apply to the true length of the nematode and not to the measured TOF. Therefore, a growth model should be applied to the ‘true’ length of the nematodes.

To address this possibility, a link between log(TOF) values of straight (where TOF is an accurate reflection of length) and curled nematodes was needed. An observed vector of log(TOF) frequencies is assumed to include measurements of a fraction of nematodes curled to various degrees. A minimum curled log(TOF) value and all log(TOF) values between the minimum and the log(TOF) corresponding to a straight nematode are equally likely is assumed. The two parameters associated with curling are then the percent of nematodes curling and the minimum log(TOF) value corresponding to the ‘most curled’ position. These two parameters are allowed to take on different values for L1s as they are loaded, as well as for different sections of the log(TOF) axis. Loaded and aspirated nematodes may pass through the flow cell differently, which may affect curling. Values for the extent and degree of curling in the cohorts of aspirated nematodes are allowed to change due to changes in *C. elegans* shape that occur during growth.

Two ‘curling’ matrices, *C_0_* and *C*, are defined with the curling parameters as follows: denote *v_0_* as the loading vector of observed log(TOF) frequencies (including curling) and *v_s0_* as the frequency vector of hypothetical lengths in log(TOF) units of the same nematodes during loading when straight. Then, *C_0_* is defined such that *v_0_ = C_0_ v_s0_*. To estimate *v_s0_*, we use an inverse matrix: *v_s0_* = *C_0_^−1^ v_0_*. Similarly for aspirated nematodes, the matrix *C^−1^* estimates the frequencies of hypothetical lengths of straight nematodes in log(TOF) units, *v_s_*, corresponding to their observed values, *v*, by *v_s_ = C^−1^v*.

The growth model was applied to the estimated log(TOF) frequencies corresponding to straight nematodes as it was for the log(EXT) data, though with transfer probabilities specific to the log(TOF) measurements. Curling and growth matrices worked together in the following way: let *v_s12k_* defined as *v_s12k_ =* 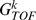 *v_s0_* denote the log(TOF) frequencies for straight *C. elegans* at aspiration time, 12 k h; then using the curling matrices predicted log(TOF) frequencies for nematodes aspirated at 12 k h, which includes possible curling, can be written as *v_12k_ = C* 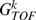 *C_0_^−1^v_0_*. The optimization proceeds as described (See Supporting Information S1 for the explicit objective function for TOF data). Growth rates in log(TOF)/h units and log(TOF) determined change points are computed the same way as the EXT measurements.

**Figure 6:**
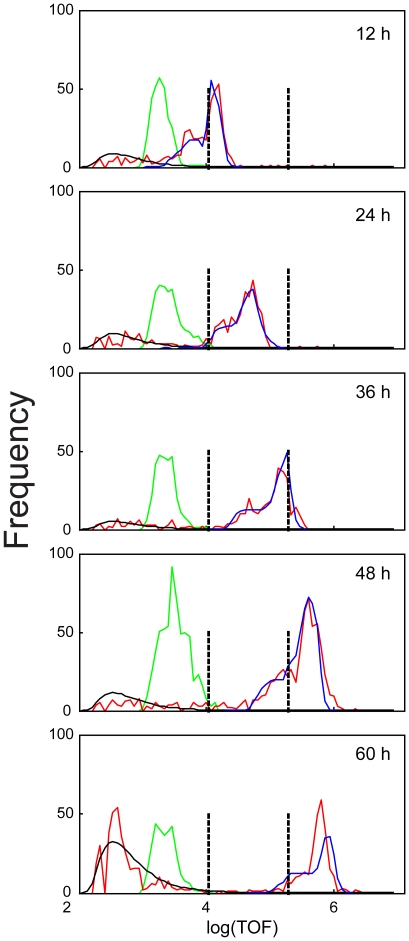
Observed and predicted frequency distributions of log(TOF) measurements at five observations times. Loading distributions for cohorts aspirated at the indicated times (*green line*) and the observed measurements (*red line*) are presented. The estimated lognormal distribution describing the extraneous measurements for each cohort (*black line*) and the predicted frequency distribution for the aspirated measurements for nematodes based on the growth model (*blue line*) are presented. The vertical lines indicate the change points at log(TOF) values of 4.035 and 5.281.

A three-valued growth rate in log(TOF) units with two change points was again found to provide an accurate fit at all observation times (Fig. 6). The expected times to growth rate change points were 7 and 34 h, compared to 11 and 51 h observed using log(EXT) values. The earlier change point might correspond to the end of the initial growth spurt after food was introduced to the starved L1s or the start of the L1/L2 molt. The initial change point was 3 h earlier than the 1^st^ change point estimated by the log(EXT) data. The second change point corresponded to the L3/L4 molt. Mostly L3s with some L4s were observed by microscopic observations at 30 h, more L4s than L3s at 32 h and mostly L4s with some L3/L4 molts occurring at 36 h.

## Discussion

The increased use of the COPAS Biosort in *C. elegans* assays is driving a need to develop quantitative analysis techniques to make full use of these measurements. The Biosort can measure up to 100 nematodes/second [9], but the output measurements (EXT and TOF) have several characteristics that make it difficult to apply standard statistical analyses. First, extraneous materials such as clumps of bacteria, discarded cuticles, or chemical precipitates that are often aspirated along with *C. elegans* are also measured by the Biosort. The measurements of extraneous material cannot always be distinguished from measurements of nematodes. This makes it difficult to estimate the numbers of aspirated nematodes at a given stage of growth. Second, although two measurements are made on each nematode (loading and aspiration) the measurements cannot be assigned to specific individual nematodes. This means growth rates for individual *C. elegans* cannot be estimated. Finally, there is evidence suggesting that a portion of the nematode population curls while passing through the flow cell. Therefore, it is not possible to know when a particular TOF measurement corresponds to a curled or a straightened nematode.

The model presented in this paper deals with these issues by using a population model to estimate frequencies of nematodes in bins defined along log(EXT) or log(TOF) axes. Growth is modeled as a random variable with a probability distribution consisting of a discrete number of probabilities. In this way, the model is a discrete convolution, similar to a continuous convolution model recently used to describe cell growth [15]. Using estimated frequency distributions, the model is able to estimate constant growth rates for log(EXT) and log(TOF) measurements, as well as the number of aspirated nematodes.

The population approach to modeling is also useful in dealing with the curling of the nematode. Ideally a nematode passes through the flow cell in a straightened position, so that TOF is highly correlated with the length of the animal. Curling disturbs this relationship producing a more variable distribution with more measurements for shorter TOFs. A quantitative analysis of TOF data that does not account for curling would underestimate nematode lengths and overestimate the natural variability in lengths.

A 48-h growth experiment showed additional low log(TOF) measurements for non-anesthetized nematodes, compared to anesthetized worms. These results are consistent with earlier reports of partial curling of a portion of nematodes affecting TOF measurements, as the relaxed, anesthetized nematodes are more successfully straightened by the faster moving core of sheath fluid in the flow cell [9]. A ‘curling’ matrix with parameter values determined by the data was used to estimate the frequency distribution of nematode lengths. Applying the growth model to the estimated length distribution accurately fit the observed log(TOF) frequency distributions after optimization. Using the model with the log(TOF) values allowed the change points in growth to be based on the ‘uncurled’ TOF values.

Three-valued growth rates were estimated for populations with respect to both log(EXT) and log(TOF) measurements. Both growth rates showed an initial high rate of growth, possibly due to the addition of food to the loading population of starved L1 *C. elegans*. During *C. elegans* development, growth rates slowed twice at the estimated change points. Expected times to these change points using log(EXT) measurements occurred at 11 and 51 h, and at 7 and 34 h for log(TOF) measurements. Using the expected times of the estimated change points, biological changes that occurred at these times could be determined by direct microscopic examination. Under the conditions of this experiment (growth at 20°C in complete K-medium with sufficient food) *C. elegans* at the earlier change points (7 and 11 h) were completing the L1/L2 molt. The 34 h TOF change point corresponded to the L3/L4 molt and *C. elegans* were non-gravid young adults at the 51 h EXT change point (see Supporting Information S1).

The results presented in this paper show that the model works well describing *C. elegans* growth from L1s to young adults. The growth rate over this interval is close enough to constant that the constraint on the predicted range of aspirated values either does not matter or accurately reflects observed behavior. To model growth from L4s to the point where adults stop growing, however, would require the model to have different structural parameters: either starting with a much larger number of smaller bins, or allowing nematodes to grow only 2, 3 or 4 bins, rather than 10, 11 or 12.

### Conclusion

Using a Markov model, *C. elegans* growth can be quantitatively analyzed using medium- and high-throughout technologies. The goals of any mathematical model are two-fold: to provide a quantitative framework for analyzing the results of experiments and to increase insight into the biological nature of the observations. A framework for the first goal was provided by defining a mathematical structure whose predictions matched the biological observations. Using estimated parameters from the model, *C. elegans* growth rates could be quantified. In addition, it was shown that *C. elegans* growth rate varied during nematode development. The points where the growth rates changed corresponded to specific physiological events: theL3/L4 molt and the start of oogenesis in the young adult *C. elegans*. The flexibility and outputs of the *C. elegans* growth model make it ideal for investigating the response of *C. elegans* to environmental agents. In a companion paper, the model was used to characterize the effects of the organophosphate insecticide chlorpyrifos of *C. elegans* growth. It should be noted that the mathematical model developed in this paper can be applied to any population that is measured more than once and for which measurements on individual objects are not linked. Thus, this model could be applied to repeated flow cytometry measurements taken on cell populations.

## Supporting Information

Supporting Information S1 Detailed description of the math involved in the model development
(0.57 MB PDF)
