# Supplementary material for: A Discrete Time Model for the Analysis of Medium-Throughput C. elegans Growth Data
Source: PLoS One. 2009 Sep 15;4(9):e7018. doi: 10.1371/journal.pone.0007018 (PMC2737628; doi:10.1371/journal.pone.0007018)

SUPPORTING INFORMATION S1

Detailed description of the math involved for the EXT observations

The mathematics of the model presented in this paper are based on grids or bins across the log-transformed ranges of log(EXT) and log(TOF). The ranges are determined by the data; [2, 7] was used for both measurements. All the data is fit to the model simultaneously, including all 5 aspirations. Since two change points were estimated, the range was divided into 3 sections: [2, kp_1], [kp_1, kp_2], [kp_2, 7].

The model predicts nematodes advance 10 – 12 bins per 12 h interval at all times with the same transfer probabilities, no matter what section of the range they are in. Thus the growth matrix, G_{EXT}, from the text retains the same format for all 5 observation times, with the number of rows equal to the total number of bins across the entire range. The number of bins nematodes are allowed to grow have corresponding transfer probabilities, p_1, p_2, and $p_3 = 1 – p_1 – p_2$. A typical growth matrix would then look as follows, with the first non-zero row being the 11th one:

$$\begin{matrix} 0 & 0 & 0 & \dots & 0 & 0 & 0 & 0 & \cdots & 0 \\ \vdots & \vdots & \vdots & \vdots & \vdots & \vdots & \vdots & \vdots & \vdots & \vdots \\ 0 & 0 & 0 & \dots & 0 & 0 & 0 & 0 & \cdots & 0 \\ p_1 & 0 & 0 & \dots & 0 & 0 & 0 & 0 & \cdots & 0 \\ p_2 & p_1 & 0 & \dots & 0 & 0 & 0 & 0 & \cdots & 0 \\ p_3 & p_2 & p_1 & \dots & 0 & 0 & 0 & 0 & \cdots & 0 \\ 0 & p_3 & p_2 & \ddots & 0 & 0 & 0 & 0 & \cdots & 0 \\ 0 & 0 & p_3 & \ddots & p_1 & 0 & 0 & 0 & \cdots & 0 \\ \vdots & \vdots & \vdots & \ddots & p_2 & p_1 & 0 & 0 & \cdots & 0 \\ 0 & 0 & 0 & 0 & p_3 & 1-p_1 & 1 & 1 & \cdots & 1 \end{matrix}$$

The rate of growth is determined by the size of the bins in terms of log(EXT) units, so that, with respect to log(EXT) units, growing over 10 large bins means a greater increase in size over 12 hrs than growing over 10 small bins. The size of the bins is determined by the number of bins in each of the sections, say, n_1, n_2 and n_3. The corresponding growth matrix has dimensions $(n_1 + n_2 + n_3) \times (n_1 + n_2 + n_3)$.

To fit the log(EXT) data, 12 parameters are estimated: the transfer probabilities (p_1 and p_2), the change points (kp_1 and kp_2), the numbers of bins in the 3 sections (n_1, n_2, and n_3), and the 5 weighting parameters nem(1), nem(2), …, nem(5) corresponding to the estimated numbers of aspirated nematodes at the 5 observation times. An additional 2 parameters are needed for the lognormal distribution of detritus. (See equation {3} in the text.).

Table A1 shows the estimated number of bins in each subrange for either experiment; Table A2 shows the transfer probabilities. The rest of the estimated parameter values were shown in the text.

Table A1. Numbers of bins/subrange

log(EXT)	log(TOF)
26 38 27	32 21 59

Table A2. Transfer probabilities

log(EXT)	Log(TOF)
0.174 0.0 0.826	0.363 0.0 0.637

Detailed description of optimization procedure

The objective function minimized over the parameters to fit the observed aspirated log(EXT) measurements is equation {4} from the text:

$$SSE = \sum_{k=1}^{5} \sum_{i=1}^{N} \left[predmix(k, x_i) - freq_{obs}(k, x_i) \right]^2$$

where k = 1, …, 5 numbers the aspiration times 12 h, …, 60 h, and i = 1, …, N numbers the bin edges which define the predictions and observations. In order for the minimization of SSE to be well-defined, the bins and edges that determine the frequencies of the observed measurements (second term above) cannot change during the optimization. But, the bins and edges that determine the predicted frequencies (first term) must be able to change during the optimization to allow growth rates with different values.

Figure A1 shows how these two conditions are reconciled. In panel A, 75 equally sized bins over the entire range are marked, with the number chosen so as to best show the features of the data. The frequencies of the observed log(EXT) measurements are taken with respect to these bins and stay the same throughout the optimization. In panel B, the change points, dividing the range into three sections are shown as vertical lines. The bins pointing the leftmost section are all the same size, but larger than those pointing the section to the right, which are larger than the bins in the last section. The optimization resulted in 26 bins to the left of the first change point, 38 bins between the change points, and 27 bins to the right of the second change point. Both the loading distribution (green) and the predicted frequency distribution for aspirated nematodes (blue) have frequencies determined by these bins. The number of rows in the growth matrix, G_{EXT}, is 91, the sum of the bin numbers.

In panel C, the 75 marked bins along the horizontal axis are again equally spaced as in panel A. The observed frequencies (red) are the same as in panel A, but re-scaled by dividing each frequency by the bin width 0.06 = (6.5 -2)/75 (Note the y-axis). The predicted frequencies in panel B are also rescaled by dividing by the bin widths corresponding to each of the three sections marked in panel B: 0.0642 = (3.669 – 2)/26 for the first section to the left; 0.0549 = (5.756-3.669)/38 for the second section; and 0.0461 = (7 – 5.756)/28 for the third section. The two curves now are on the same scale and are super-imposed in panel C. The blue curve still consists of 91 connected points, but can be evaluated at the 75 bin edge points shown in panel C

using the matlab interp1 function. These interpolated values are used in equation {3} of the text, together with the lognormal distribution also evaluated at the 75 edge points. The final figure in panel D is found by multiplying all frequencies by the bin width of the 75 bins shown in panel D.

Detailed description of the model for TOF, including the explicit objective function

A growth model for log(TOF) observations is more difficult to formulate, because growth affects the length of the nematodes, which is not directly observed. The observed TOF measures the length of the interruption of the laser as the nematode passes, possibly in a bent or curled position. A curled position would result in a lower TOF reading for a nematode, while the same nematode in a straightened out position would result in a higher log(TOF) reading, more reflective of the nematode's length. Since anesthesia paralyzes the muscles of the nematodes, it was thought likely that anesthetized nematodes would be straighter than non-anesthetized nematodes as they pass by the laser. Figure 5 comparing log(TOF) histograms of anesthetized and non-anesthetized nematodes is consistent with this hypothesis.

For this reason, we estimated the distributions of log(TOF) values corresponding to straightened out positions of nematodes (more reflective of the length distribution) from the observed distributions of log(TOF) measurements, that includes curled nematodes. The growth model is applied to the modified log(TOF) values, that more closely reflect nematode lengths. Then, with these predictions in hand, we reversed the conversion to obtain an estimated distribution of log(TOF), including curling, that could be directly compared to the observed distributions at 12, 24, …, 60 h.

To define the 'curling matrices', that link log(TOF) values including curling with log(TOF) values without curling, we start with an imagined vector of log(TOF) frequencies for straightened out nematodes. Since curling can only reduce the log(TOF) measurement, not lengthen it, we assume a minimum curled log(TOF) value and then assume that all log(TOF) values between the minimum and the value corresponding to a stretched out nematode are equally likely. Two parameters associated with curling are then: the minimum log(TOF) value corresponding the 'most curled' position, and the percent of nematodes curling.

Given the two parameters associated with curling defined above, we consider the following matrix as a building block with n rows and m columns.

$$C_{k(n\ x\ m)} = \begin{bmatrix} 1 & pc_k & pc_k/2 & \cdots & 0 & 0 \\ 0 & 1-pc_k & pc_k/2 & \cdots & pc_k/7 & 0 \\ 0 & 0 & 1-pc_k & \vdots & \vdots & \vdots \\ 0 & 0 & 0 & \ddots & pc_k/7 & pc_k/7 \\ \vdots & \vdots & \vdots & \vdots & 1-pc_k & pc_k/7 \\ 0 & 0 & 0 & 0 & 0 & 1-pc_k \end{bmatrix} \qquad \{1\}$$

Here pc_k is the fraction of nematodes in a curled or bent position. If the matrix, C_k, is multiplied on the right by log(TOF) frequencies corresponding to straight nematodes, the diagonal element determines the fraction of nematodes that are not curled and hence don't get moved to a bin for

smaller log(TOF) measurements. The elements of the matrix above the diagonal correspond to bins with lower log(TOF) values. Suppose a straight nematode attains its maximum log(TOF) value in the 27th bin. The smallest log(TOF) value corresponding to maximum curling, might be 0.75 x max log(TOF). Then the elements in the 27th column from row numbers 20 (20.25 = 0.75 x 27) to 26 are all non-zero with equal probabilities that sum to pc_k (ie, $pc_k/7$). Note that the elements of $C_{k(n \times m)}$ are completely determined by two parameters: maximum attenuation due to curling (0.75 in the example) and the percent of nematodes curling (pc_k). These parameters are determined as part of the optimization process.

The curling matrix has the same number of rows and columns as the total number of variably-sized bins used in the optimization (corresponding to the bins shown in Figure A1, panel B), but the values of the two curling parameters (minimum log(TOF) value due to curling and the percent curled worms) are allowed to differ between the sections defined by change points (See text) as well as between loading and aspiration. Thus, two curling matrices are needed (loading and aspiration), each defined as block matrices with blocks of the format shown in {1} and with row and column numbers equal to the number of bins in each section. Thus, since all the loading data has log(TOF) values corresponding to the first section, we can defined C_{load} as follows:

$$C_{load\,(n_1+n_2+n_3)x(n_1+n_2+n_3)} = \begin{bmatrix} C_{0(n_1 x n_1)} & 0 & 0 \\ 0 & I_{(n_2 x n_2)} & 0 \\ 0 & 0 & I_{(n_3 x n_3)} \end{bmatrix}$$

where $C_{0(n1 \times n1)}$ is defined as in [5] with corresponding pc_0 and a minimum log(TOF) values and n_i refers to the number of bins in the ith section from the left.

Similarly, the aspiration matrix looks the following:

$$C_{asp(n_1+n_2+n_3)x(n_1+n_2+n_3)} = \begin{bmatrix} C_{1(n_1 x n_{11})} & 0 & 0 \\ 0 & C_{2(n_2 x n_2)} & 0 \\ 0 & 0 & C_{3(n_3 x n_3)} \end{bmatrix}$$

With parameters pc_1, pc_2, pc_3 and 3 more minimum log(TOF) values.

The above matrices are used to define the equation for predicted aspirated log(TOF) measurements analogous to equation {3} in the text. For the kth observation point at t = 12 k hours,

$$predmix(t,\vec{x}) = (asp(k) - nem(k)) * \log normal(\vec{x},\mu,\sigma) + \frac{nem(k)}{load(k)} C_{asp} G^t C_{load}^{-1} v_0(\vec{x})$$

Again, the values denoted by these terms were interpolated over the grid of fixed and equally sized bins across the log(TOF) range. We used 75 bins for the fixed grid. Note that the 5 values

of nem(12k) are those estimated using the log(EXT) measurements and are not re-estimated using the log(TOF) measurements. Other than the estimates of the aspirated nematodes, fitting the log(TOF) data requires estimating analogous parameters to those estimated using the log(EXT) (transition probabilities, change points and the number of bins in each section of the range), adding the above-defined curling parameters (minimum log(TOF) value due to curling and percent of nematodes curled).

The estimated values of these parameters are shown in Table A3. Curling nematodes may reduce their log(TOF) readings to between 0.67 and 0.93 of the maximum log(TOF). There does not seem to be much of a trend. Similarly, the percent of nematodes curling varies between 32% and 95%, again without much trend.

Tables A3. Percent not straight (for estimated (TOF))

	**% curling**	**Minimum fraction of length**
loading	32%	0.93
section 1	95%	0.88
section 2	48%	0.67
section 3	56%	0.67

FIGURE LEGEND

Figure A1. *Panel A* shows the observed frequencies with respect to the grid formed by 75 bins of equal width. The data corresponds to that taken at 12 h. *Panel B* shows the predicted frequencies with respect to variable bin sizes: the vertical lines mark the change points (also shown as Figure 1). Bin sizes to the left of the change points are larger than those to the right. *Panel C* superimposes the predicted curve from panel B onto the panel A, dividing each curve by the width of the corresponding bins. The bins shown are the same as in panel A, with equal widths. The two curves are now on the same scale. Thus, the predicted histogram (blue line) can be read at the edge points of the fixed bins with equal widths. *Panel D* shows both graphs from panel C, re-scaled by multiplying by the width of the equally sized bins. The lognormal density was evaluated at the bin edge points and the weighting factors shown in Equation {3} of the text were used.

Figure A2. Growth rates with respect to log(EXT) (*upper panel*) and log(TOF) (*lower panel*). Times at which growth rates changed are shown as dotted lines. Microscopic observations made around change points are shown as text

Figure 1-Appdx

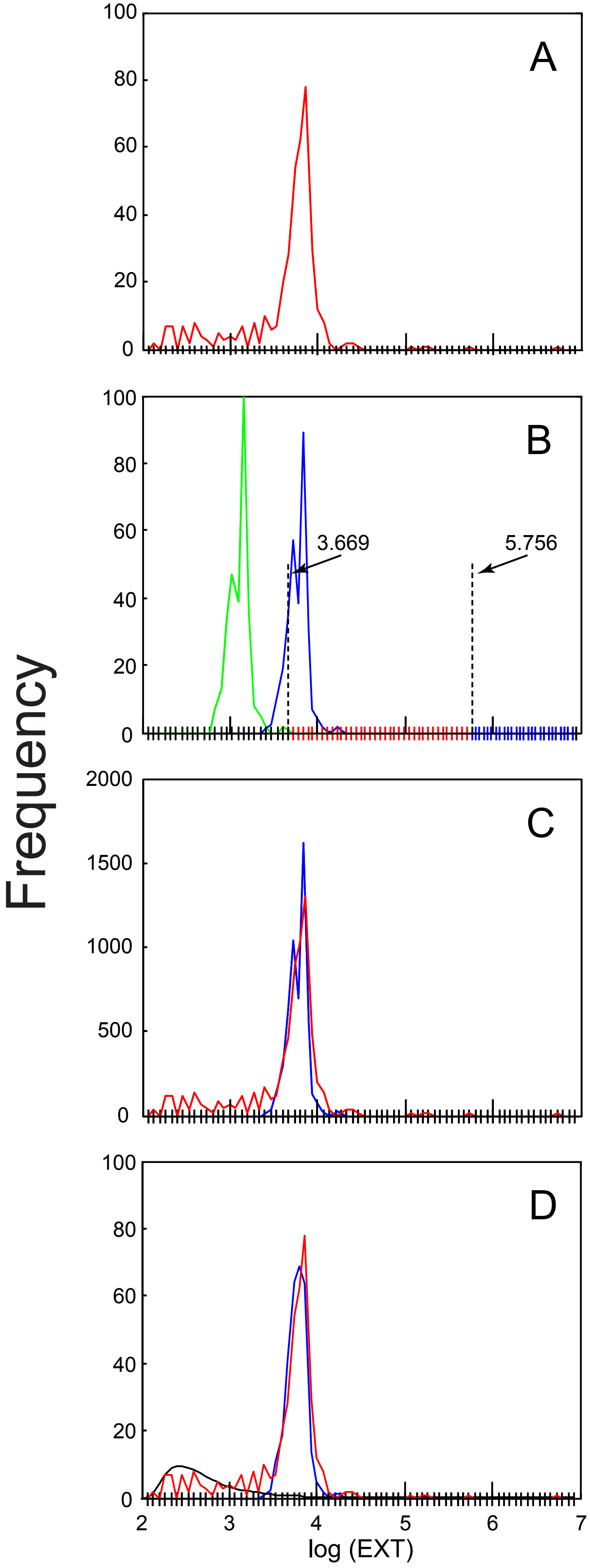

Figure 2 - Appdx

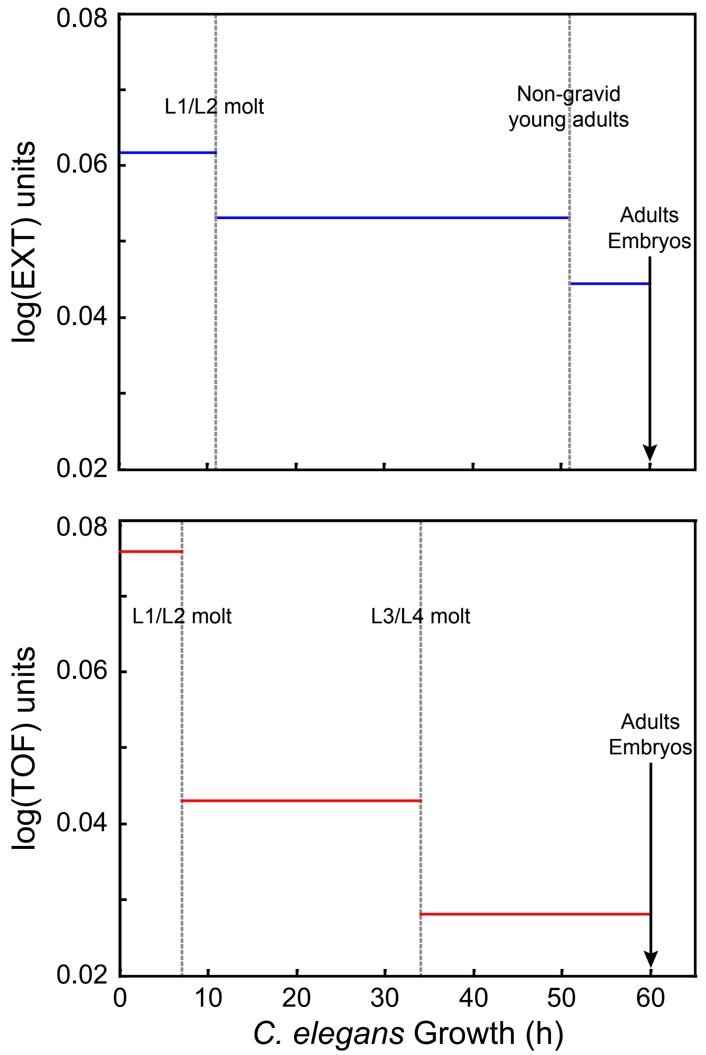

Supplement: Supporting Information S1 — Detailed description of the math involved in the model development (0.57 MB PDF) [file pone.0007018.s001.pdf]
